# Ocular adverse effects of Topiramate: Two case reports

**DOI:** 10.4103/0253-7613.45156

**Published:** 2008

**Authors:** Ananya Mandal, Suparna Chatterjee, Sagarmay Bose, Gautam Ganguly

**Affiliations:** Department of Pharmacology, Institute of Postgraduate Medical Education and Research, Kolkata, Department of Neuromedicine (former attachment), India; 1 Department of Neuromedicine, Bangur Institute of Neurosciences and Psychiatry, Kolkata, India

**Keywords:** Adverse effects, ocular, topiramate

## Abstract

Topiramate, an antiepileptic drug is reported to cause various ocular adverse effects like acute onset myopia, glaucoma. Visual field defect is an uncommon, serious treatment emergent adverse effect. We are reporting two cases of suspected topiramate induced visual field defects.

Both the cases were on topiramate for more than 6 months as add-on therapy at daily doses ranging from 100-150mg. The presenting complaints were insidious onset visual disturbances. Diagnosis was based of temporal association with drug intake, clinical examination and investigations. Automated perimetry revealed bilateral superior quadrantic and arcuate field defects in the two cases respectively. Marked improvement with drug dechallenge was noted which was also corroborated by perimetry. Using Naranjo's ADR Probability Scale, both cases revealed a “probable” association with topiramate. This report intends to improve awareness amongst clinicians to facilitate early diagnosis and intervention.

## Introduction

Topiramate has been approved for use as an add-on therapy or monotherapy for resistant partial seizures and generalized seizures. Its other uses include migraine prophylaxis, bipolar and post-traumatic stress disorders, and neuralgias.[1] Its antiepileptic activity is attributed mainly to sodium channel blockade, activation of GABA_A_ receptors and weak anti-carbonic anhydrase activity. The common adverse effects of topiramate are somnolence, fatigue, psychomotor slowing and cognitive dysfunction.[1,2] Its ocular adverse effects include acute angle closure glaucoma, ocular pain, headache, hyperemia, mydriasis, uveitis, visual field defects, acute onset myopia, suprachoroidal effusions, blepharospasm, oculogyric crisis, retinal hemorrhage and scleritis.[2]

Acute angle closure glaucoma with raised intra ocular pressure is a common ocular adverse effect of topiramate. However, visual field defect with normal intraocular pressure is a rare but serious treatment emergent adverse event of topiramate. In this case series, we report two cases of suspected topiramate-induced reversible bilateral visual field defects. A MEDLINE search on the above subject shows that there are only two reports of similar reversible visual field defects with topiramate usage.[3,4]

## Case Reports

### Case 1

A 22-year-old male presented at the epilepsy clinic with repeated episodes of partial seizure with secondary generalization. On screening, the Magnetic Resonance Imaging (MRI) and Computerized Tomography (CT) scan of the brain revealed a ring enhancing lesion in the right parietal region, with perilesional edema. Phenytoin 300 mg/day was prescribed initially, with subsequent addition of topiramate (100 mg/day) for effective seizure control. He was also given zolpidem (5 mg/day) and clonazepam (1 mg/day) for a period of three months.

After seven months of therapy with topiramate, he complained of insidious onset of visual difficulty. Detailed ophthalmologic examination revealed normal fundus and intraocular pressure in both eyes. A refractive error, myopia, was detected (R/E -1.0 D and L/E -1.0 D). His complaints persisted, despite correction of refractive error. Routine hematological and biochemical profiles and serum phenytoin level were found to be within normal limits. An MRI of the brain showed marked regression of both the initial lesion and reduction of perilesional edema.

Automated perimetry (Humphrey Visual Field Analyzer, Peripheral 30-2 Threshold Test Strategy) showed peripheral constriction limited to superior quadrant in the right eye and the nasal half of the visual field in the left eye. The reliability of the test was deemed acceptable and the percentage of false positive and false negative was near 0% in both the eyes (acceptable limits being less than 33%).[5]

**Figure 1:**
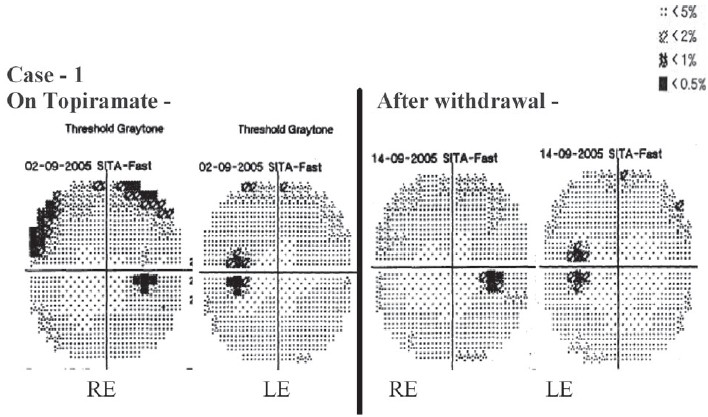
Case 1: Automated Perimetry (Central 30-2 Threshold test strategy) report of both eyes while the patient was on Topiramate, and after withdrawal (RE - Right Eye; LE - Left Eye)

Topiramate was withdrawn gradually, within six weeks. Perimetry, after four weeks of drug withdrawal, showed that both eyes had threshold sensitivity within normal limits, with definite reduction in the field loss [Figure 1]. A provisional diagnosis of suspected topiramate-induced visual field defect was made on the basis of temporal association, marked improvement with dechallenge, previous reports of association of such field defects with topiramate[3,4] and exclusion of other ocular and non-ocular diseases like glaucoma, space occupying lesion in the pituitary fossa or optic cortex. Although the patient was also taking phenytoin, clonazepam and zolpidem, literature search revealed no published cases of visual defects associated with clonazepam and zolpidem. Although phenytoin can cause ocular ADRs, the visual complaints and repeat perimetry report showed marked improvement even when phenytoin, clonazepam and zolpidem were continued and only topiramate was withdrawn. The ring enhancing lesion was located in the right parietal region; therefore this lesion could not be the cause of bilateral visual field defects, due to its anatomical location. Using the Naranjo's ADR probability score, it was concluded that this case revealed a probable association with topiramate.[6]

### Case 2

A 21-year-old female patient of generalized tonic clonic epilepsy was on carbamazepine (400 mg/day) for more than five years. Due to inadequate seizure control, carbamazepine dose was uptitrated and topiramate (50 mg/day) added. Seizure control was achieved with carbamazepine (1200 mg/day), topiramate (150 mg/day) and clobazam (15 mg/day). Routine hematological and biochemical profiles were within normal limits.

The patient complained of difficulty in peripheral vision after about a year of topiramate usage and detailed ocular examination revealed normal fundus, intraocular pressure. An MRI report of the brain was also normal. Due to persistent visual complaints, automated perimetry (Humphrey Visual Field Analyzer Central 24-2 threshold test strategy) was done, which showed bilateral superior arcuate defects in both eyes. The reliability of the test was acceptable and the percentage of false positive and false negative was near 0% in both the eyes (acceptable limits being less than 33%).

**Figure 2:**
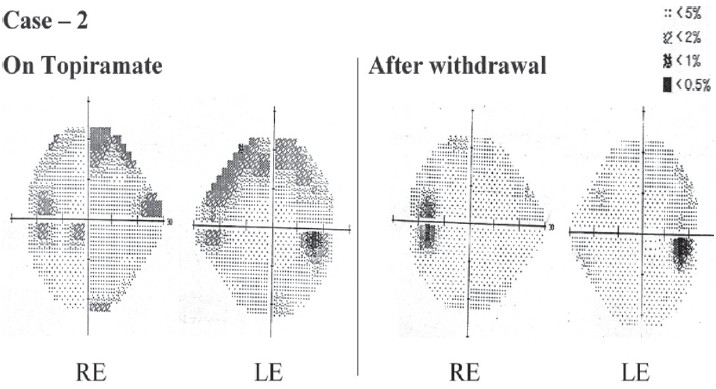
Case 2: Automated Perimetry (Central 24-2 Threshold test strategy) results of both eyes while the patient was on Topiramate, and after withdrawal (RE - Right Eye; LE - Left Eye)

The dose of topiramate was gradually tapered and finally withdrawn. Perimetry after four weeks of drug withdrawal showed significant improvement of the field defects in both eyes [Figure 2]. The patient was on concomitant carbamazepine, which may cause visual field defects. However, in this case, there was improvement in vision after withdrawal of topiramate, although carbamazepine and clobazam were continued. This reduced the possibility of carbamazepine being the suspect drug. However, it needs to be investigated whether concurrent administrations of drugs, which have effects on the GABA ergic pathway, add to the risk. Causality assessment using the Naranjo's ADR probability score revealed a probable association.[5]

## Discussion

A visual field defect is partial loss of the usual field of vision, that maybe central or peripheral. The common causes of visual field defects include glaucoma, retinal detachment and central retinal artery occlusion. Although rare, some drugs implicated in the causation of visual field defects are isotretinoin, chloroquine, vigabatrin, tiagabine, gabapentine, diazepam, phenytoin, carbamazepine and topiramate.[6,7] Of the two published reports of topiramate-induced field defects, the first case developed right incongruous homonymous hemianopia with topiramate usage for migraine prophylaxis, and the second case developed homonymous hemianopia after intake of topiramate for 12 weeks. Partial recovery after drug discontinuation was noted in both the cases.[3,4]

Topiramate has several pharmacokinetics drug interactions with other anti-epileptic drugs, namely phenytoin, carbamazepine, valproic acid, phenobarbitone, primidone and lamotrigine. While lamotrigine can increase the plasma concentration of topiramate, phenytoin and carbamazepine may decrease it. Therefore, in the two cases that we are reporting, the occurrence of visual adverse effects cannot be explained on the basis of pharmacokinetic drug interactions with phenytoin and carbamazepine, since these drugs may decrease the plasma concentration of topiramate. However, the possibility of pharmacodynamic interactions cannot be ruled out.

The exact mechanism of drug-induced ocular field defects is not well understood. Vigabatrin- induced ocular field defects that are on record have led to the hypothesis that increased persistence of GABA in the retina; lateral geniculate nucleus and the visual cortex could be an underlying cause.[7,8] A recently published animal study, that evaluated the effects of chronic administration of topiramate in rabbits, has revealed that there was a significant reduction of the retinal function demonstrated by the reduced b-wave amplitude in the full-field Electroretinogram. Immunohistochemical changes characterized by a severe accumulation of GABA in the inner retina were observed.[9] The retinal dysfunction and the morphological changes indicate that topiramate may damage the retina, similar to vigabatrin.

## Conclusion

Since most anticonvulsant drugs are indicated for long term usage, their safety profile demands close monitoring. Spontaneous ADR reporting is an important tool for detecting such reactions. This case series intends to improve awareness amongst clinicians and patients about such serious ocular ADRs, to facilitate early diagnosis and intervention.

## References

[1] Lyseng-Williamson KA, Yang LP (2007). Topiramate: A review of its use in the treatment of epilepsy. Drugs.

[2] National registry of drug-induced ocular side effects http://www.piodr.sterling.net.

[3] Foroozan R, Buono LM (2003). Foggy visual field defect. Surv Ophthalmol.

[4] Asensio-Sanchez VM, Torreblanca-Aguera B, Martinez-Calvo S, Calvo MJ, Rodriguez R (2006). Severe ocular side effects with Topamax. Arch Soc Esp Oftalmol.

[5] Naranjo CA, Busto U, Sellers EM, Sandor P, Ruiz I, Roberts EA (1981). A method for estimating the probability of adverse drug reactions. Clin Pharmacol Ther.

[6] Stefan H, Bernatik J, Knorr J (1999). Visual Field defects due to antiepileptic drugs. Nervenarzt.

[7] Sihota R, Tandon R, Sihota R, Tandon R (2003). Diseases of the optic nerve. Parson's diseases of the eye.

[8] Hilton EJ, Hosking SL, Betts T (2004). The effect of antiepileptic drugs on visual performance. Seizure.

[9] Kjellstrom S, Bruun A, Isaksson B, Eriksson T, Andreasson S, Ponjavic V (2006). Retinal function and histopathology in rabbits treated with Topiramate. Doc Ophthalmol.

